# Oxygen Radical Scavenger Activity, EPR, NMR, Molecular Mechanics and Extended-Hückel Molecular Orbital Investigation of the Bis(Piroxicam)Copper(II) Complex

**DOI:** 10.1155/MBD.1995.43

**Published:** 1995

**Authors:** Renzo Cini, Rebecca Pogni, Riccardo Basosi, Alessandro Donati, Claudio Rossi, Luciano Sabadini, Libertario Rollo, Sauro Lorenzini, Renata Gelli, Roberto Marcolongo

**Affiliations:** 1 Department of Chemistry University of Siena Pian dei Mantellini 44 Siena 1-53100 Italy; 2 Institute of Reumathology University of Siena Via dei Tufi 3 Siena 1-53100 Italy

## Abstract

The oxygen radical scavenger activity (ORSA) of [Cu^II^(Pir)_2_] (HPir = Piroxicam = 4-hydroxy -2- methyl -*N*-2- pyridyl -2*H*- 1,2-benzothiazine -3- carboxamide 1,1-dioxide) was determined by chemiluminescence of samples obtained by mixing human neutrophils (from healthy subjects) and [Cu^II^(Pir)_2_(DMF)_2_] (DMF = N,N -dimethylformammide) in DMSO/GLY/PBS (2:1:2, v/v) solution (DMSO = dimethylsulfoxide, GLY = 1,2,3-propantriol, PBS = Dulbecco’s buffer salt solution). The ratio of the residual radicals, for the HPir (1.02·10^−4^M) and [Cu^II^(Pir)_2_(DMF)_2_] (1.08·10^−5^M)/HPir (8.01·10−^−5^M) systems was higher than 12 (not stimulated) [excess of piroxicam was added (Cu/Pir molar ratio ≈1:10) in order to have most of the metal complexed as bischelate]. In contrast, the ratio of residual radicals for the CuCl_2_ (1.00·10^−5^M) and [Cu^II^(Pir)_2_(DMF)_2_] (1.08·10^−5^M)/Hpir (8.01·10^−5^M)system was 5. The [Cu^II^(Pir)_2_] compound is therefore a stronger radical scavenger than either HPir or CuCl_2_. A molecular mechanics (MM) analysis of the gas phase structures of neutral HPir, its zwitterionic (HPir^+-^) and anionic (Pir^-^) forms, and some Cu^II^-piroxicam complexes based on X-ray structures allowed calculation of force constants. The most stable structure for HPir has a ZZZ conformation similar to that found in the Cu^II^ (and Cd^II^  complexes) in the solid state as well as in the gas phase. The structure is stabilized by a strong H bond which involves the N(amide)-H and O(enolic) groups. The MM simulation for the [Cu^II^(Pir)_2_(DMF)_2_]  complex showed that two high repulsive intramolecular contacts exist between a pyridyl hydrogen atom of one Pir^-^  molecule with the O donor of the other ligand. These interactions activate a transition toward a *pseudo*-tetrahedral geometry, in the case the apical ligands are removed. On refluxing a suspension of [Cu^II^(Pir)_2_(DMF)_2_] in acetone a brown microcystalline solid with the Cu(Pir)_2_·0.5DMF stoichiometry was in fact prepared. ^13^C spin-lattice relaxation rates of neutral, zwitterionic and anionic piroxicam, in DMSO solution are explained by the thermal equilibrium between the three most stable structures of the three forms, thus confirming the high quality of the force field. The EPR spectrum of [Cu^II^(Pir)_2_(DMF)_2_] (DMSO/GLY, 2:1, v/v, 298 and 110 K) agrees with a N2O2+O2 *pseudo*-octahedral coordination geometry. The EPR spectrum of [Cu^II^(Pir)_2_·0.5DMF agrees with a *pseudo*-tetrahedral coordination geometry. The parameters extracted from the room temperature spectra of the solution phases are in agreement with the data reported for powder and frozen solutions. The extended-Hückel calculations on minimum energy structures of [Cu^II^(Pir)_2_(DMF)_2_] and [Cu^II^(Pir)_2_] (square planar) revealed that the HOMOs have a relevant character of d_x_^2^−y^2^. On the other hand the HOMO of a computer generated structure for [Cu^II^(Pir)_2_] (*pseudo*-tetrahedral) has a relevant character of d_xy_ atomic orbital. A d_xy_ orbital is better suited to allow a dπ-pπ interaction to the O_2_^-^ anion. Therefore this work shows that the anti-inflammatory activity of piroxicam could be due in part to the formation of [Cu^II^(Pir)_2_] chelates, which can exert a SOD-like activity.


### OXYGEN RADICAL SCAVENGER ACTIVITY, EPR, NMR, MOLECULAR MECHANICS AND EXTENDED-HÜCKEL MOLECULAR ORBITAL INVESTIGATION OF THE BIS(PIROXICAM)COPPER(II) COMPLEX

Renzo Cini*[1], Rebecca Pogni[1], Riccardo Basosi[1], Alessandro Donati[1], Claudio Rossi[1], Luciano Sabadini[2], Libertario Rollo[2], Sauro Lorenzini[2], Renata Gelli[2], and Roberto Marcolongo[2]

[1] Department of Chemistry, University of Siena, Pian dei Mantellini 44, I-53100 Siena
[2] Institute of Reumathology, University of Siena, Via dei Tufi 3, I-53100 Siena, Italy.

### Abstract

The oxygen radical scavenger activity (ORSA) of $[Cu^{II}(Pir)_2]$ (HPir = Piroxicam = 4-hydroxy -2- methyl -*N*-2- pyridyl -2*H*- 1,2-benzothiazine -3- carboxamide 1,1-dioxide) was determined by chemiluminescence of samples obtained by mixing human neutrophils (from healthy subjects) and $[Cu^{II}(Pir)_2(DMF)_2]$ (DMF = N,N -dimethylformammide) in DMSO/GLY/PBS (2:1:2, v/v) solution (DMSO = dimethylsulfoxide, GLY = 1,2,3-propantriol, PBS = Dulbecco's buffer salt solution). The ratio of the residual radicals, for the HPir ($1.02 \cdot 10^{-4}$ M) and $[Cu^{II}(Pir)_2(DMF)_2]$ ($1.08 \cdot 10^{-5}$ M)/HPir ($8.01 \cdot 10^{-5}$ M) systems was higher than 12 (not stimulated) [excess of piroxicam was added (Cu/Pir molar ratio ≈1:10) in order to have most of the metal complexed as bischelate]. In contrast, the ratio of residual radicals for the $CuCl_2$ ($1.00 \cdot 10^{-5}$ M) and $[Cu^{II}(Pir)_2(DMF)_2]$ ($1.08 \cdot 10^{-5}$ M)/HPir ($8.01 \cdot 10^{-5}$ M) systems was 5. The $[Cu^{II}(Pir)_2]$ compound is therefore a stronger radical scavenger than either HPir or $CuCl_2$. A molecular mechanics (MM) analysis of the gas phase structures of neutral HPir, its zwitterionic ($HPir^{+-}$) and anionic ($Pir^-$) forms, and some $Cu^{II}$-piroxicam complexes based on X-ray structures allowed calculation of force constants. The most stable structure for HPir has a ZZZ conformation similar to that found in the $Cu^{II}$ (and $Cd^{II}$ complexes) in the solid state as well as in the gas phase. The structure is stabilized by a strong H bond which involves the N(amide)-H and O(enolic) groups. The MM simulation for the $[Cu^{II}(Pir)_2(DMF)_2]$ complex showed that two high repulsive intramolecular contacts exist between a pyridyl hydrogen atom of one $Pir^-$ molecule with the O donor of the other ligand. These interactions activate a transition toward a *pseudo*-tetrahedral geometry, in the case the apical ligands are removed. On refluxing a suspension of $[Cu^{II}(Pir)_2(DMF)_2]$ in acetone a brown microcystalline solid with the $Cu(Pir)_2 \cdot 0.5DMF$ stoichiometry was in fact prepared. $^{13}C$ spin-lattice relaxation rates of neutral, zwitterionic and anionic piroxicam, in DMSO solution are explained by the thermal equilibrium between the three most stable structures of the three forms, thus confirming the high quality of the force field. The EPR spectrum of $[Cu^{II}(Pir)_2(DMF)_2]$ (DMSO/GLY, 2:1, v/v, 298 and 110 K) agrees with a N2O2+O2 *pseudo*-octahedral coordination geometry. The EPR spectrum of $Cu^{II}(Pir)_2 \cdot 0.5DMF$ agrees with a *pseudo*-tetrahedral coordination geometry. The parameters extracted from the room temperature spectra of the solution phases are in agreement with the data reported for powder and frozen solutions. The extended-Hückel calculations on minimum energy structures of $[Cu^{II}(Pir)_2(DMF)_2]$ and $[Cu^{II}(Pir)_2]$ (square planar) revealed that the HOMOs have a relevant character of $d_{x2-y2}$. On the other hand the HOMO of a computer generated structure for $[Cu^{II}(Pir)_2]$ (*pseudo*-tetrahedral) has a relevant character of $d_{xy}$ atomic orbital. A $d_{xy}$ orbital is better suited to allow a $d\pi$-$p\pi$ interaction to the $O_2^-$

---

† Author to whom the correspondence should be addressed anion. Therefore this work shows that the anti-inflammatory activity of piroxicam could be due in part to the formation of $[Cu^{II}(Pir)_2]$ chelates, which can exert a SOD-like activity.

### Introduction

Copper plays important roles in the humans (it is the third most abundant d-block element, after iron and zinc) as well as in all the other living organisms. This metal is present in several types of enzymes which are involved in oxydo-reduction processes, oxygen transport, copper storage, etc.[1]

Free radicals as $O_2^{-}$, $OH^{\cdot}$, $RO_2^{\cdot}$ (R = H or an organic group), are released in important physiological and pathological phenomena such as cell respiration, peroxidation of membrane lipids, exposure to radiation.[2] Probably the most efficient superoxide scavenger of the enzyme superoxide dismutase (SOD) family is (Cu,Zn)-SOD which has been found in animals, plants and primitive organisms.[3,4]

The X-ray structure of bovine erythrocyte (Cu,Zn)-SOD and computer modeling have led to a proposed catalytic mechanism of the $O_2^{-}$ dismutation in which the substrate links covalently to the $Cu^{II}$ centers (it is oxidized to $O_2$, first step).[3]

Piroxicam (feldene, Pfizer; Scheme 1, HPir) is a powerful anti-inflammatory agent.

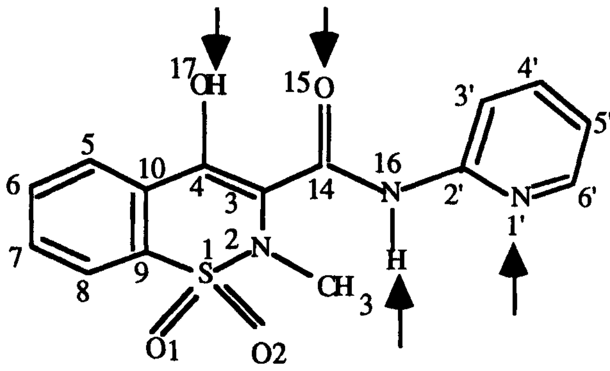

Scheme 1. Neutral piroxicam, HPir, in the 4,16-EZE conformation.

It has been shown that copper complexes of anti-inflammatory anti-arthritic drugs are more active than their parent compounds.[5] Furthermore, toxicological studies revealed that anti-inflammatory copper complexes are less toxic than inorganic forms of copper.[5] These facts have led to the hypothesis that copper complexes of non-steroidal anti-inflammatory drugs are the active species *in vivo*.[5,6]

On the basis of such arguments, we reasoned that recently prepared and structurally characterized M(II)-piroxicam (M = Fe, Co, Ni, Cu, Zn and Cd) chelates[7] could have (Cu,Zn)-SOD like activity.

Measurements of the reactivity of $[Cu^{II}(Pir)_2(DMF)_2]$ with $O_2^{-}$ and other oxygen radicals, such as HO·, HOO·, as well as with singlet-$O_2$, revealed that, on a molar basis, the $Cu^{II}$ derivative is a far better scavenger than piroxicam.

Electron paramagnetic (EPR, X-band) and nuclear magnetic ($^1H$, $^{13}C$ NMR) resonance spectroscopies, as well as molecular mechanics (MM) and extended-Hückel molecular orbital (EHMO) methods, were used to understand the structure of the coordination sphere in solution and the mechanism of (Cu,Zn)-SOD like activity of the $Cu^{II}$-piroxicam complexes.

### Results and Discussion

Oxygen radical scavenger activity (ORSA).

Owing to the insolubility of $[Cu^{II}(Pir)_2(DMF)_2]$ in water and to the labile nature of the $Cu^{II}$-L bonds it was necessary to set up a suitable solvent system, and *ad hoc* conditions to have only $Cu^{II}$-species of a defined stoichiometry.

The DMSO/GLY/PBS (GLY = 1,2,3-propantriol; PBS = Dulbecco's buffer salt solution) mixture (see Experimental) dissolve $[Cu^{II}(Pir)_2(DMF)_2]$, $CuCl_2{\cdot}H_2O$ and HPir at least up to $1.1{\cdot}10^{-5}$, $1.1{\cdot}10^{-5}$ and $1.1{\cdot}10^{-4}$ M, respectively, in the range 20-40°C. The solutions are stable for at least a week, and allow the human neutrophils to survive for at least 3 hours at 37°C (mixtures containing fractions of the PBS component higher than ca. 40% (v/v) produced some precipitation of $Cu^{II}$-species).

The main dissolving component is DMSO; however, mixtures of DMSO/GLY (2:1, v/v) are good solvents for $[Cu^{II}(Pir)_2(DMF)_2]$.†

The PBS component fraction, for the present work, was the maximum allowed by the homogeneous system. This was done to make the conditions as close as possible to the physiological environment and to allow the measurement of the scavenger activity.§

The ORSA values for $[Cu^{II}(Pir)_2(DMF)_2]$/HPir, HPir and $CuCl_2$ in DMSO/GLY/PBS can be obtained from integrated chemiluminescence signals (ICS) reported in Table I.

Table I. Integrated chemiluminescence signals (ICS, x$10^6$/c.p.m.). The values reported are the mean of data collected for three sets of experiments. Each system was measured twice in every experiment. Corrections for dilution effects due to the addition of zymosan (ZYM, ≈ 10%) were always applied. The estimated error for all the data is 6%. The values in parenthesis are relevant to polymorphonucleates (PMNs) from another subject.

| PBS | PBS (ZYM) | PBS/ DMSO/ GLY [2/2/1] | PBS/ DMSO/ GLY [2/2/1] (ZYM) | HPir | HPir (ZYM) | $[Cu(Pir)_2(DMF)_2]$/ HPir | $[Cu(Pir)_2(DMF)_2]$/ HPir (ZYM) | $CuCl_2$ |
|---|---|---|---|---|---|---|---|---|
| 283 | 580 | 11.5 | 19.4 | 7.8(16.4) | 7.6(16.7) | 0.4(1.3) | 0.9(1.4) | 2.1 |

The ratio of ICS values for HPir and $[Cu^{II}(Pir)_2(DMF)_2]$/HPir, and $CuCl_2$ and $[Cu^{II}(Pir)_2(DMF)_2]$/HPir ranges between 12.6 and 19.5 (unstimulated) and between 8.4 and 11.9 (stimulated), and 5.0

† It has to be noted that DMSO/GLY mixtures were used as solvents to test the anti-inflammatory activity of the $Cu^{II}$-salicylate species dermally applied to rats.[8]

§ As the Pir/Cu molar ratio of the solution is ca. 10:1, it can be reasonably assumed that all the metal is linked to the $Pir^-$ anion in the form of $Cu^{II}(Pir)_2$, on the basis of the value of $\beta_2$ (10.23, 35°C, $KNO_3$ 0.1M, 50% dioxan) for $Cu^{II}$(2-pyridylacetate)$_2$:[9a] the free $Cu^{2+}{}_{(solv)}$ should not be higher than 1% ($10^{-7}$ M). It can also be estimated that the formation of $Cu^{II}$-chloride and -phosphate species is negligible for the conditions used in the present work (conc. of $Cl^-$ and total phosphate 0.06 and 0.004 M, respectively; $K(Cu^{II}X)$ = 1.18 and 3.2 $mol^{-1}$.L for X = $Cl^-$ and $HPO_4^{2-}$, respectively [9a]). The ligating ability of LUM should be lower than that of 2-aminobenzoic acid (for the latter $logK_1(CuX)$ = 4.25, I = 0, 25°C)[9b] and it is two order of magnitude lower than that of 2-pyridylacetate[9a] and that of piroxicam. Phthalate, the compound obtained by oxidation of LUM, is even a weaker ligand than LUM itself ($logK_1(CuX)$ = 3.75, I = 0.1, 25°C).[9c] Therefore the formation of $Cu^{II}$-LUM and -phthalate species can be neglected. It has to pointed out that the EPR spectrum of $[Cu^{II}(Pir)_2(DMF)_2]$/HPir/LUM (1, 10, and 10 mM, respectively) in DMSO/GLY (110 K) is superimposable with that recorded for the $[Cu^{II}(Pir)_2(DMF)_2]$/HPir system. Owing to the low concentration of DMF (ca. $2{\cdot}10^{-5}$ M) it is reasonable that most of the $Cu^{II}$ ion contained in the solution is not linked to the amide molecules. The coordination spheres (for *pseudo*-octahedral or *pseudo*-square pyramidal species) can be completed by solv (solv = $H_2O$, DMSO, GLY) molecules. As a consequence it is assumed that the ORSA values measured in this work are attributable to $[Cu^{II}(Pir)_2]$, $[Cu^{II}(Pir)_2(solv)]$ or $[Cu^{II}(Pir)_2(solv)_2]$ species (see below, for the analysis of possible active coordination geometries).

(respectively). These data show that ORSA for $[Cu^{II}(Pir)_2(DMF)_2]$ is much higher than ORSA for HPir, and it is higher than ORSA for copper(II) chloride, too.

Furthermore two other experimental facts have to be noted and analyzed. First, the DMSO and GLY solvents have their own ORSA activity (see Table I). The ratio of ICS values for PBS/DMSO/GLY (2/2/1, v/v/v) and PBS mixtures is $3.3 \cdot 10^{-2}$ (stimulated); however, the ICS values for the solvent system are large enough to allow accurate measurements of scavenger activity. Second, the stimulating effect of ZYM in the presence of just HPir does not exist (ratio of ICS values ≈ 1) whereas it is detectable for the $[Cu^{II}(Pir)_2(DMF)_2]$/HPir system (the ratio of ICS values ranges from 1.1 to 2.3). It has to be noted that HPir gave inhibition against some activators of receptor-mediated rat leukocyte aggregation.[10] It is reasonable that a type of PMN receptor - HPir interaction inhibits also the oxygen radical stimulation activity of ZYM. However such a type of mechanism is much less efficient when copper(II) species are present. The affinity of copper(II) species for PMNs is able to delete (at least in part) the inhibitory effect of HPir.

### Molecular Mechanics

*Neutral Piroxicam, HPir.* Rotations around the C(3)-C(14), C(14)-N(16) and N(16)-C(2') bond axes (Scheme 1) of the solid state structure,[11a] produced a total of 43 reliable independent conformations. Structure I ($E_{Tot}$ = 39.52 kcal/mol) has a EZE[12] conformation (Scheme 1) and nicely superimposes with the experimental structure (RMS = 0.089 Å for S,O, and N atoms). A *H-bond* interaction links the O(15) and O(17) atoms. The conformation of structure II (38.38) is EZZ. The $E_{Tot}$(I)-$E_{Tot}$(II) difference (1.14 kcal/mol) is mostly due to the repulsive van der Waals interaction between O(15) and H(3') in I. III (37.82, ZZE) and IV (Figure 1a, 36.70, ZZZ) are stabilized by the strong *H-bond* which links the N(16) and O(17) atoms. V (37.60, ZZZ) has the methyl group cis to the $(S)O_2$ oxygen atoms; it is destabilized in comparison with IV because the repulsive interaction between O(15) and the methyl goup hydrogen atoms.

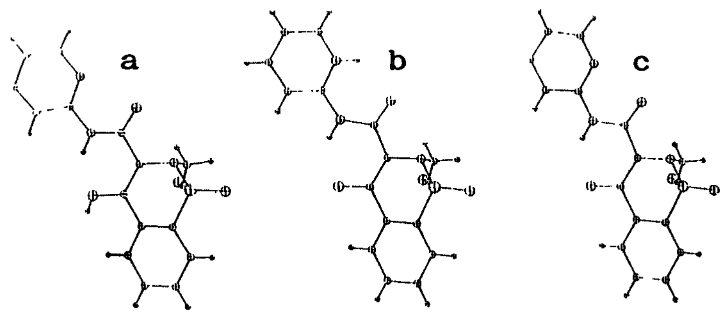

Figure 1. ORTEP[26] drawings of the most stable structures of the: a) neutral (HPir), b) zwitterionic ($HPir^{+-}$), and c) anionic ($Pir^-$) molecules.

*Zwitterionic Piroxicam, $HPir^{+-}$.* The conformational analysis for the zwitterionic form was carried out with a procedure similar to that above reported for the neutral molecule. It produced 36 reliable conformations from the solid state structure;[13] four more stable energy minimized structures were finally obtained. Structure I (Figure 1b, 34.53, ZZZ) is the most stable one and it has a good agreement with the experimental solid state structure (RMS = 0.105 Å, on S,O,N atoms). It is stabilized by two strong *H-bonds* involving the N(1') and O(15), and the N(16) and O(17) couples of atoms. Other structures are: II (35.80, ZZE), III (35.96, EZZ), and IV (37.28, EZE).

*Anionic Piroxicam, Pir$^-$.* The four more stable structures of the Pir$^-$ form are: I (Figure 1c, 37.80, ZZZ), *H-bond* N(16)···O(17); II (39.20, EZZ); III (39.90, ZZE); IV (41.30, EZE).

It has to be pointed out that the most stable structure for neutral HPir, zwitterionic HPir$^{+-}$ and anionic Pir$^-$ has a ZZZ conformation, whereas the solid state structures of neutral HPir have a EZE conformation.[11] This discrepancy may be explained by the intermolecular N(16)···O(1) hydrogen bonds which stabilizes the solid state structure.

The analysis of the NMR data (see below) is in good agreement with the MM results for the HPir, HPir$^{+-}$ and Pir$^-$ molecules. This prompted us to apply the force field used for the Pir$^-$ moieties, to the [Cu$^{II}$(Pir)$_2$(DMF)$_2$] molecule (see below). Furthermore, the present NMR investigation and a recently published NMR work on piroxicam[14] suggest that both in chloroform solution (preponderance of neutral HPir, and low solvation effects) and in polar solvents such as DMF, DMSO, DMSO/$H_2O$ (high solvation effects, and presence of both neutral HPir and zwitterionic HPir$^{+-}$), the ZZZ conformation is prominent. This type of ligand conformation facilitates the N2O2 chelation of the $Cu^{2+}$ cation, in the solution phase.

*[Cu$^{II}$(Pir)$_2$(DMF)$_2$].* The energy minimized structure of the complex molecule with an equatorial N2O2 coordination set (Figure 2, 77.56, ZZZ) nicely superimposes with the X-ray structure[7] (RMS = 0.08 Å, based on Cu, S, N, and O atoms).

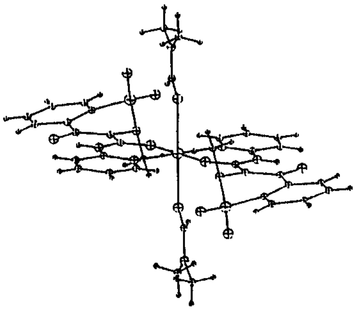

Figure 2. ORTEP[26] drawings of the most stable structure of the [Cu$^{II}$(Pir)$_2$(DMF)$_2$] complex molecule with the N2O2 donor set.

The structure of the solid state and gas phase [Cu$^{II}$(Pir)$_2$(DMF)$_2$] molecule has two H(6)'···O(15) repulsive intramolecular short contacts (3.51 kJ/mol, 2.34 Å). The two O(15)-N(16)-C(1')-N(1')-C(6') moieties of the complex molecule are forced to be coplanar by the apical amide ligands which form weak Cu-O bonds. On removing the axial ligand (e.g. by refluxing a mixture of [Cu$^{II}$(Pir)$_2$(DMF)$_2$] in acetone, see Experimental) a brown powder is obtained. This is reversibly transformed back into the green solid when mixed with DMF.†

On the basis of the EPR data (see below) it is argued that the brown crystalline powder Cu$^{II}$(Pir)$_2$·0.5DMF consists mostly of distorted tetrahedral Cu$^{II}$(Pir)$_2$.

NMR spectroscopy.

The experimental carbon spin-lattice relaxation rates for a 0.15M piroxicam solution in DMSO and the dipolar contribution to the relaxation (see Experimental) are reported in Table II.

† The reduction of the Cu$^{II}$-complex to Cu$^{I}$ species (see reaction of anhydrous $CuCl_2$ with acetone to produce CuCl)[15] is excluded by: (1) the existence of an unpaired electron located on the metal, as detected by EPR spectroscopy (see below); (2) the fast reversibility to the green species when treated with DMF or DMSO.

Table II. Experimental relaxation parameters of a 0.15 M DMSO solution of piroxicam at 298K.

| Carbon n. | $\delta$ (ppm) | $R_{1C}$ ($s^{-1}$) | NOE (BB) | $\chi^{DD}$ | $R_1^{DD}$ ($s^{-1}$) |
|---|---|---|---|---|---|
| 14 | 166.81 | 0.15 | 1.58 | 0.80 | 0.12 |
| 4 | 160.01 | 0.18 | 1.60 | 0.81 | 0.15 |
| 2' | 150.00 | 0.19 | 1.55 | 0.79 | 0.15 |
| 6' | 145.45 | 1.90 | 1.95 | 0.98 | 1.88 |
| 4' | 139.90 | 2.10 | 1.96 | 0.99 | 2.09 |
| 9 | 134.63 | 0.11 | 1.60 | 0.81 | 0.09 |
| 6 | 133.12 | 2.80 | 1.97 | 1.00 | 2.80 |
| 7 | 132.71 | 3.40 | 1.96 | 0.99 | 3.38 |
| 10 | 129.31 | 0.14 | 1.46 | 0.74 | 0.10 |
| 5 | 126.54 | 2.60 | 1.95 | 0.98 | 2.58 |
| 8 | 123.88 | 2.60 | 1.97 | 1.00 | 2.60 |
| 5' | 120.00 | 3.75 | 1.95 | 0.98 | 3.73 |
| 3' | 116.37 | 1.60 | 1.94 | 0.98 | 1.58 |
| 3 | 110.75 | 0.08 | 1.40 | 0.71 | 0.05 |

In order to analyse the experimental carbon relaxation rates on the basis of the equilibria between stable conformations the theoretical spin-lattice relaxation rate $R_1^T$ of the most significant quaternary carbons of piroxicam was computed by taking into account all the intramolecular proton-carbon dipolar interactions within a cut off distance of 3.5 Å and by using the $R_1^T = \Sigma p_i R_1^i$ relationship ($p_i$ is the fractional population of each conformation and $R_1^i$ is the theoretical contribution related to a specific interaction). The dipolar theoretical terms related to each $^1H$-$^{13}C$ interaction for five quaternary carbons of piroxicam as well as the proton-carbon distances of the nuclei involved in the dipolar interactions are reported in Table III.

Table III. Comparison of theoretical carbon spin-lattice rate $R^T_1$ with the dipolar contribution to carbon spin-lattice relaxation rate of a 0.15M piroxicam solution. The theoretical $R^T_1$ was calculated as a sum of contributions arising from the three most stable conformations.

| Interactions | | I [1]$p_I$ = 0.729 | | II [1]$p_{II}$ = 0.161 | | III [1]$p_{III}$ = 0.111 | | |
|---|---|---|---|---|---|---|---|---|
| | [2]r | [3]$R^I_1$ | [2]r | [3]$R^{II}_1$ | [2]r | [3]$R^{III}_1$ | [4]$R^T_1$ | [5]$R^{DD}_1$ |
| H17 | 3.005 | 0.006 | 3.015 | 0.006 | 3.175 | 0.005 | | |
| [6]C3 | | | | | | | 0.02 | 0.05 |
| H16 | 2.491 | 0.018 | 2.492 | 0.018 | 2.476 | 0.019 | | |
| | | | | | | | | |
| H5 | 2.664 | 0.012 | 2.651 | 0.014 | 2.658 | 0.014 | | |
| [6]C4 H16 | 2.445 | 0.020 | 2.404 | 0.022 | 2.432 | 0.021 | 0.15 | 0.15 |
| H17 | 1.804 | 0.122 | 1.806 | 0.122 | 1.958 | 0.075 | | |
| | | | | | | | | |
| H5 | 2.005 | 0.065 | 2.004 | 0.068 | 2.005 | 0.066 | | |
| [6]C10 | | | | | | | 0.08 | 0.10 |
| H17 | 2.465 | 0.019 | 2.443 | 0.020 | 2.522 | 0.017 | | |

| | | | | | | | | | |
|---|---|---|---|---|---|---|---|---|---|
| | | | | | | | | | |
| | H16 | 1.853 | 0.104 | 1.855 | 0.104 | 1.846 | 0.105 | | |
| [6]$C_{14}$ | | | | | | | | 0.11 | 0.12 |
| | H17 | 3.535 | 0.003 | 3.514 | 0.004 | 3.714 | 0.002 | | |
| | | | | | | | | | |
| | H16 | 1.939 | 0.061 | 1.036 | 0.062 | 1.931 | 0.063 | | |
| [7]C2' | H3' | 1.902 | 0.068 | 1.903 | 0.068 | 1.905 | 0.049 | 0.12 | 0.15 |
| | [8]H1' | 2.161 | 0.032 | 2.004 | 0.050 | 2.010 | 0.049 | | |

[1] Fractional populations of each stable conformation. [2] Proton-carbon distance. [3] Dipolar relaxation contributions referred to the specific interactions. [4] Theoretical carbon spin-lattice relaxation rate calculated as: $R_1^T = \sum \rho_i R_1$. [5] Dipolar contribution to the experimental $R_1$ determined as: $R_1^{DD} = R_1 e^{\chi^{DD}}$. [6] Dipolar proton-carbon interaction modulated by a $\tau_c = 1.9 \times 10^{-10}$. [7] Dipolar proton-carbon interaction modulated by a $\tau_c = 9 \times 10^{-11}$. [8] Contribution due to the zwitterionic form (33% abundance).

A good agreement between experimental and theoretical values was usually found (some discrepancy was observed for the C(3) carbon). It has to be pointed out that the contribution of the zwitterionic $HPir^{+-}$ form (33%)[14] was also considered in the calculation of $R_1^T$ for C(2'). A similar approach brought also to a good agreement between observed $R_1^{DD}$ and computed $R_1^T$ values for $Pir^-$. Furthermore, the calculated atomic charges of both HPir and $Pir^-$ (Extended-Huckel method (see below) applied to the most stable structures) describes with accuracy the chemical shift properties observed for the two species (see supplementary material available from the authors).

These results confirm that the previously proposed equilibrium between neutral HPir and zwitterionic $HPir^{+-}$ [14] and the present MM analysis are basically correct.

EPR spectroscopy

The EPR spectrum of $[Cu^{II}(Pir)_2(DMF)_2]$/HPir in DMSO/GLY (110 K, Figure 3a, Table IV) shows the typical pattern of pseudo-octahedral (tetragonal elongation) copper(II) complexes in which three of the expected four hyperfine transitions in the parallel region (low-field range) are visible whereas the fourth component ($M_I$ = +3/2) is masked by the overlap with $A_\perp$ features in the high-field end of the spectrum.

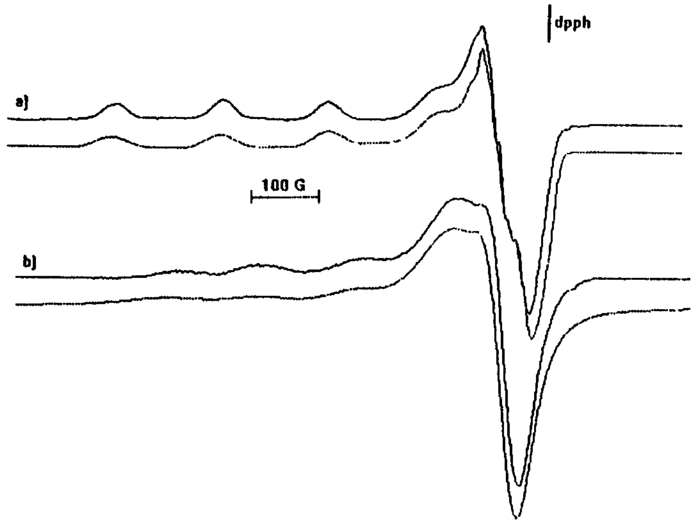

Figure 3. X-band EPR spectra of $[Cu^{II}(Pir)_2(DMF)_2]$/HPir (Cu:piroxicam molar ratio, 1:10) in DMSO/GLY: a) 110 K, ν = 9.6239MHz, and b) 298 K, ν = 9.6217MHz. The respective simulations are also reported; the magnetic parameters are listed in Table IV.

Table IV. X-band Spin Hamiltonian EPR parameters.

| Species | $g_z$ | $g_v$ | $g_x$ | Cu $A_z^a$ | Cu $A_y^a$ | Cu $A_x^a$ | N $A_{//}$ | N $A_\perp$ | $\tau_c$ $10^{-12}$ s |
|---|---|---|---|---|---|---|---|---|---|
| $[Cu(Pir)_2(DMF)_2]$ /HPir in DMSO/GLY 110 K | 2.290 | 2.060 | 2.060 | 500.0 | 29.0 | 29.0 | 45.0 | 37.0 | |
| Idem 298 K | 2.290 | 2.060 | 2.060 | 500.0 | 29.0 | 29.0 | 45.0 | 37.0 | 833 |
| $Cu(Pir)_2 \cdot 0.5DMF \cdot$ Powder, 110 K | 2.237 | 2.070 | 2.058 | 532.0 | 58.0 | 43.0 | 41.0 | 37.0 | |

[a]All hyperfine coupling constants are in MHz. Error limits: $g_z = \pm 0.003$, $g_y = g_x = \pm 0.004$, Cu ($A_z$) = ± 9 MHz, Cu($A_{x,y}$) = ±12 MHz, N(A) = ±1 MHz

The room temperature spectrum of slow tumbling $[Cu^{II}(Pir)_2(DMF)_2]$/HPir in DMSO/GLY, (Figure 3b, Table IV) is typical of a quasi-immobilized species for the high viscosity of the solution ($\tau_c$ = 833 x$10^{-12}$ sec).

The EPR spectra of the $Cu^{II}$-complex used for ORSA studies were simulated by using computer programs for: a) rigid limit, and b) slow motion conditions and taking into account the simultaneous presence of the two copper isotopes ($^{63}Cu$ (69%) and $^{65}Cu$ (31%); nuclear spin 3/2; ratio of nuclear moments for $^{63}Cu$ and $^{65}Cu$, 1.07). Since the geometry of the coordination sphere is crucial for defining ORSA of metal complexes, the EPR spectrum of the $Cu^{II}(Pir)_2 \cdot 0.5DMF$ powder (Table IV) was also deeply investigated. It clearly reveals a distorted tetrahedral geometry.[16]

Extended-Hückel Molecular Orbital Analysis and $Cu^{II}$-$O_2^-$ Interaction

The HOMO in the $[Cu^{II}(Pir)_2(DMF)_2]$, $[Cu^{II}(Pir)_2(DMF)]$ (square pyramidal), and $[Cu^{II}(Pir)_2]$ (square planar) molecules has a relatively high Cu($d_{x^2-y^2}$) character (Table V, main contributions from donor atom orbitals: O15($p_y$), N1'($p_x$)). These results are in agreement with previously reported theoretical investigations on $Cu^{II}$-complexes.[17]

Table V. HOMOs for the molecules studied through the Extended-Hückel method. Coefficients of atomic orbitals higher than 0.20 are reported. The origin of the coordinate system is on the Cu atom for the copper complexes and on O1 for the $O_2^-$ anion. For $[Cu^{II}(Pir)_2(DMF)_2]$ the z axis points toward the DMF oxygen donor; the x and y axes point toward N1'B and O15B donors, respectively. (A and B refers to the two $Pir^-$ ligands). For tetrahedral $[Cu^{II}(Pir)_2]$ the reference system is skeched in Scheme 2b.

| Molecule | Atomic orbital coefficients | | |
|---|---|---|---|
| $[Cu^{II}(Pir)_2(DMF)_2]$ | 0.402[$p_y$(O15A)] | -0.305[$p_x$(N1'A)] | 0.244[$p_y$(O17A)] |
| | -0.402[$p_y$(O15B)] | 0.305[$p_x$(N1'B)] | -0.244[$p_y$(O17B)] |
| | 0.444[$d_{x^2-y^2}$(Cu)] | | |
| | | | |
| $[Cu^{II}(Pir)_2]$ tetrah. | 0.276[$p_x$(N2A)] | -0.280[$p_y$(C3A)] | 0.348[$p_z$(O15A)] |
| | -0.369[$p_x$(O17A)] | 0.304[$p_z$(O17A)] | -0.206[$p_z$(C3B)] |
| | 0.253[$p_z$(O15B)] | 0.204[$p_z$(O17B)] | -0.210[$d_{x^2-y^2}$(Cu)] |

| | | | |
|---|---|---|---|
| | | | |
| $O_2^-$ | -0.577[$p_y$(O1)] | 0.489[$p_z$(O1)] | 0.577[$p_y$(O2)] |
| | -0.489[$p_z$(O2)] | | |

The eventual formation of a $Cu^{II}$-$O_2^-$ bonding interaction is highly hindered by the presence of the apical (amide) ligands. Furthermore the interaction of the metal complexes with the $O_2^-$ ion is also not permitted by the symmetry of the HOMOs of the two species.

For example, the $O_2^-$ anion can approach the metal center of the square planar $[Cu^{II}(Pir)_2]$ at the apical sites, but owing to the high $p_y$ and $p_z$ character of the oxygen orbitals (x is the direction of the superoxide O-O bond axis, Scheme 2a, Table V) of the HOMO of $O_2^-$,

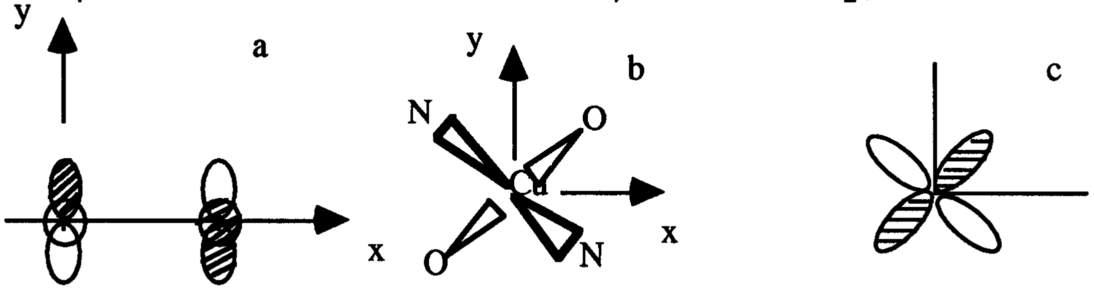

Scheme 2. a) HOMO of the superoxide anion $O_2^-$. The z axis points toward the observer; b) the coordination sphere for the tetrahedral $Cu^{II}(Pir)_2$ molecule (the metal sits on the origin, the N and O atoms have positive and negative values of the z coordinate, respectively); c) the contribution of the Cu($d_{xy}$) atomic orbital to the HOMO of the complex molecule.

only a weak bent  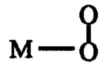 arrangment with a dδ-pπ overlap is possible for the $[Cu^{II}(Pir)_2]$ (square planar) complex. On the other hand the HOMO of the tetrahedral structure of $[Cu^{II}(Pir)_2]$ (Scheme 2b,c, Table V) has some Cu($d_{xy}$) character (main contributions from ligand atom orbitals: O15, O17, C3, and N2). The $O_2^-$ ion and the complex molecule could thus interact via a pπ-dπ overlap.

A distorted tetrahedral geometry could be a convenient compromise for a suitable Cu-$O_2^-$ bond and weak $O_2^-\cdots Pir^-$ repulsions. Some distortion from pure tetrahedral geometry for a $d^9$ configuration is also demanded by the Jahn-Teller theorem.[17b]

It has to be pointed out that the coordination geometry of $Cu^{II}$ in (Cu,Zn)-SOD, as obtained from X-Ray diffraction, is *pseudo*-tetrahedral,[3] and computer simulated docking investigations showed that the $O_2^-$ ion and the $CN^-$ enzyme inhibitor fitted one and both the available sites around the metal center, respectively.[3]

We infer that the anti-inflammatory activity of piroxicam passes through the $O_2^-$ (and other oxygen radicals) scavenger activity of its $Cu^{II}$-complexes.

Owing to the local high concentration of piroxicam in the cell (during the drug supply) and to the low amount of free $Cu^{2+}$ ion, the presence of $[Cu^{II}(Pir)_2]$ (*pseudo*-tetrahedral coordination geometry) as the active species is highly probable.

On the other hand the neutral molecule $[Cu^{II}(Pir)_2]$ can posses higher membrane permeability than charged particles, such as $[Cu^{II}(H_2O)_4]^{2+}$, $[Cu^{II}(Pir)(H_2O)_2]^+$.

### Experimental Part

Materials.

Analytical grade dimethylsulfoxide (DMSO), deuterated dimethylsulfoxide-$d_6$, 99.8% D (DMSO-$d_6$), analytical grade $CuCl_2\cdot 2H_2O$, acetone and N,N-dimethylformamide were purchased from

(Roma). Dulbecco's phosphate buffered salt (modified, without Mg & Ca) solution (PBS) was obtained by dissolving 1 tablet (Flow Laboratories, U.K.) in water (100 mL). Glycerol was purchased from Carlo Erba (Milano).

Polymorphprep (PMP), Zymosan, Luminol (LUM), and $NH_4Cl$ (analytical grade) were from Nycomed Pharma (Oslo) and Sigma (St. Louis). Polymorphonucleates were from peripheral blood of healthy human subjects.

Methods

*Synthesis of $[Cu^{II}(Pir)_2(DMF)_2]$.* The materials and the procedure used for the synthesis of the compound are those reported in Ref. 7.

*Synthesis of $Cu^{II}(Pir)_2 \cdot 0.5DMF$.* 50 mg of $[Cu^{II}(Pir)_2(DMF)_2]$ were mixed with 20 mL of acetone in a 100 mL Erlenmayer flask. The mixture was boiled under stirring until the volume of the solvent reduced to about 5 mL. The brown suspension was then added of acetone and concentrated (by heating) four times. The final mixture (5 mL) was cooled to room temperature and the brown solid was filtered off and dried in the air for 48 hours. Anal. Calcd. for $C_{30}H_{24}N_6CuO_8S_2 \cdot 0.5(C_3H_7NO)$ ($M_w$ = 760.8): N, 11.97; S, 8.43. Found: N, 11.7; S, 8.27.

*EPR Spectroscopy.* All the solutions of $[Cu^{II}(Pir)_2(DMF)_2]$ in DMF, DMSO, and DMSO/GLY (2:1, v/v) used for the EPR measurements contained $1 \cdot 10^{-3}$ M of the complex compound. The solution in DMSO/GLY contained also free HPir ($0.8 \cdot 10^{-2}$ M). Each sample was contained in a 1.0x1.2 mm quartz tube.

X-band EPR spectra were obtained with a Bruker 200D SRC X-band spectrometer using a high sensitivity Bruker ER 4108 TMH cavity. Microwave frequencies were measured through a XL Microwave Model 3120 counter. Magnetic field was calibrated with a MJ-140 magnetometer by Jagmar (Poland). The temperature was controlled by using a Bruker variable temperature unit ER 4111 VT. The spectrometer was interfaced with a PS/2 Technical Instruments Hardware computer and the data acquired using the EPR data system CS-EPR produced by Stelar Inc. (Mede, Italy).

The spectra were simulated through the COSMOS (low motion),[18] and CUSIMNE (rigid limit)[19] programs, implemented on a Compaq Deskpro 486/50L personal computer with a 8-MByte memory and a 50-MHz clock.

*NMR Spectroscopy.* A Varian XL-200 spectrometer was used for recording $^1H$ and $^{13}C$ spectra. Carbon spin-lattice relaxation rates were obtained by using the inversion recovery $(\pi-\tau-\pi/2\text{-}t)_n$ pulse sequence. $R_1$ values were calculated by computer-fitting of the relaxation curves. NOE values were determined through the equation: NOE = $(I_z\text{-}I_0)/I_0$ ($I_z$ and $I_0$ represent the peak intensities measured under continuous and gated proton decoupling, respectively). A 5% experimental error was estimated for $R_1$ and NOE measurements. The fractional dipolar contribution to the carbon spin-lattice relaxation rates, $\chi^{DD}$, was determined by comparing the experimental NOE with the theoretical expected value for $^{13}C$ nuclei totally relaxed by $^1H\text{-}^{13}C$ dipolar interaction. The dipolar contribution to the experimental carbon-spin lattice relaxation rate was calculated by using the following equation: $R_1^{DD} = \chi^{DD}.R_{1exp}$. The correlation times modulating the C-H magnetic interactions were computed from the $R_1^{DD}$ values by using standard C-H distances (1.08 Å) and the equations reported in Ref 20. All NMR chemical shift values were referred to internal DMSO-$d_6$.

*Oxygen Radical Scavenger Activity.* A stock solution of the $[Cu^{II}(Pir)_2(DMF)_2]$ complex was prepared by dissolving 9.4 mg of the complex ($1.08 \cdot 10^{-2}$ mmol) and 26.5 mg of HPir ($8.01 \cdot 10^{-2}$ mmol) in 10 mL of DMSO. 0.1 mL of the stock solution was mixed with 4 mL of DMSO, 2 mL of GLY and with PBS up to a total volume of 10 mL (20°C); final analytical concentrations: $C_{Cu} = 1.08 \cdot 10^{-5}$ M, $C_{Pir} = 10.17 \cdot 10^{-5}$ M.

A stock solution of HPir was obtained by mixing 26.5 mg of the drug ($8.01 \cdot 10^{-2}$ mmol) with 10 mL of DMSO. 0.1 mL of the stock solution was diluted to 10 mL with the DMSO/GLY/PBS solvent system by following the procedure above reported for the solution of $[Cu^{II}(Pir)_2(DMF)_2]$; final analytical concentration: $C_{Pir} = 10.17 \cdot 10^{-5}$ M.

A stock solution of $CuCl_2 \cdot 2H_2O$ was prepared by dissolving 4.26 mg of the compound ($2.50 \cdot 10^{-2}$ mmol) in 25 mL of DMSO. 0.1 mL of the stock solution was mixed with DMSO, GLY and PBS by using the same procedure listed above for $[Cu^{II}(Pir)_2(DMF)_2]$ and HPir; final analytical concentration: $C_{Cu} = 1.00 \cdot 10^{-5}$ M.

The oxygen free radical (produced by human PMNs) scavenger activity of $[Cu^{II}(Pir)_2(DMF)_2]$/HPir, HPir and $CuCl_2$ in DMSO/GLY/PBS (see above) was measured through the chemiluminescence technique, by using a chemiluminometer Berthold Multi-Biolumat LB 9505C.

Preparation of PMN. PMN were separated from blood by using the following procedure. 5 mL of blood were mixed with 3.5 mL of PMP in a 10 mL test tube. The mixture was centrifugated at 450÷500g (30 mins, 20±2°C). The PMN contained in the lower ring were rinsed with a PBS solution. The residual erythrocytes were then distroyed by adding a solution of ammonium chloride (0.83 g/100 mL of water). The purified PMN were then added to the sample solution.

Preparation of the LUM solution. 2 mg of LUM were dissolved in NaOH 0.01M (10 mL). The proper amount of the stock solution was added to the sample solution in order to get a concentration of $1 \cdot 10^{-4}$ M.

Opsonization of the cells by Zymosan. 5 mg of ZYM were mixed with 4.5 mL of PBS solution and with 0.5 mL of plasm (obtained during the separation of PMN from blood through centrifugation, see above; plasm is the lightest fraction). The mixture was incubated at 37°C for 30 mins and then centrifugated at 900g for 10 mins. The pellet of ZYM was rinsed twice with PBS and then suspended in PBS (0.5 mL).

Chemiluminescence measurements. The samples containing the PMNs ($10^6$/mL), LUM, the drugs and eventually the stimulator (ZYM) in the DMSO/GLY/PBS solvent were tested (at 37°C) through the chemiluminometer and the intesity of the light emitted was recorded for a period of 40 mins. The integrated signal was computed via the computer program Berthold LB 9505 C Version 4.07.

*Molecular Mechanics.* The strain energies of the free ligand and the metal-complex molecules were computed as the sums: $E_{Tot} = E_b + E_\theta + E_\phi + E_{nb} + E_{hb}$; $E'_{Tot} = E_{Tot} + E_\varepsilon$ ($E_b$, $E_\theta$, $E_\phi$, $E_{nb}$, $E_{hb}$ e $E_\varepsilon$ are the bond length deformation, the valence angle deformation, the torsional angle deformation, the non bonding interaction, the hydrogen bond interaction and the electrostatic contributions, respectively). The force field used was MM2.[21] Modification and extension of the force field was necessary in order to take into account the interactions between the metal center and the donor atoms. The proper force field parameters were obtained via a *trial and error* procedure which brought to an excellent agreement between calculated and observed structures. The new force field parameters used in this study are reported in Table VI. They are in good agreement with those previously used in molecular mechanics studies on other metal complexes with N and/or O donor atoms.[22]

Table VI. Details of the force field for bonds involving the metal center.[a]

| bond | $R_0$, Å | $K_b$, mdyn·Å$^{-1}$ |
|---|---|---|
| Cu-O(Pir) | 1.94 | 1.05 |
| Cu-N(Pir) | 2.08 | 0.89 |
| Cu-O(Amide) | 2.46 | 0.70 |

| angle | $\theta_0$, deg | $K_\theta$, mdyn·rad$^{-1}$ |
|---|---|---|
| O(Pir)-Cu-N | 90 | 0.15 |
| O(Pir)-Cu-O(Pir) | 180 | 0.15 |
| O(Amide)-Cu-O(Pir) | 90 | 0.15 |
| O(Amide)-Cu-N | 90 | 0.15 |
| O(Amide)-Cu-O(Amide) | 180 | 0.15 |
| Cu-O(Pir)-C | 128 | 0.55 |
| Cu-O(Amide)-C | 128 | 0.37 |
| Cu-N-C | 120 | 0.50 |

[a] All the torsional angles A-B-C-D with A or D = Cu were assigned torsional terms equal 0; those with B or C = Cu were fixed at the experimental solid state values.

The total energy ($E_{Tot}$ or $E'_{Tot}$) was minimized with the block diagonal matrix Newton-Raphson method until the root mean square value (RMS) of the first derivative vector was less than 0.01 kJ/Å. The starting structures were those found for the solid state via single crystal X-ray diffraction for HPir (Ref. 11a, see also 11b) and its zwitterionic form,[13] and for the $[Cu^{II}(Pir)_2(DMF)_2]$ complex.[7]

The calculations were carried out by using the MacroModel (MMOD) package version 3.0[23] implemented on a VAX 6600 computer with a graphic ouput on an Evans & Sutherland PS390.

The atomic-charge calculations were performed by using the extended-Hückel method via the ICONC&INPUTC[24] program (see below). List of selected atomic charges are available as supplementary material.

As the $E_\varepsilon$ contribution did not produce any appreciable improvement in the MM analysis, all the calculations reported in this work do not take into account the $E_\varepsilon$ term. This approximation is often applied for calculations of metal-complex molecules.[22a,b]

*Extended-Hückel Calculations.*- The molecular orbital calculations were performed through ICONC&INPUTC[24] program implemented on a VAX 6600 computer. The parameters used were those reported in Table VII, with distance-dependent weighted Wolfsberg-Helmholz formula.[25]

Table VII. Parameters used in Extended-Hückel calculations (included in ICONC&INPUTC[24]).

| Atom | Orbital | $H_{ii}$(eV) | $\xi_1$ | $\xi_2$ | $C_1$ | $C_2$ |
|---|---|---|---|---|---|---|
| | | | | | | |
| Cu | 3d | -14.00 | 5.95 | 2.3 | 0.5934 | 0.5744 |
| | 4s | -11.40 | 2.20 | | | |
| | 4p | -6.06 | 2.20 | | | |
| | | | | | | |
| S | 3s | -20.5 | 2.28 | | | |
| | 3p | -11.40 | 1.817 | | | |
| | | | | | | |
| O | 2s | -28.20 | 2.575 | | | |
| | 2p | -12.40 | 2.275 | | | |
| | | | | | | |
| N | 2s | -26.00 | 2.14 | | | |
| | 2p | -13.40 | 1.95 | | | |

| | | | | | | |
|---|---|---|---|---|---|---|
| C | 2s | -21.40 | 1.71 | | | |
| | 2p | -11.40 | 1.625 | | | |
| | | | | | | |
| H | 1s | -13.60 | 1.30 | | | |

The self-consistent charge iteration calculation on the Cu atom was performed on the complex molecule. The VSIE (valence state ionization energy) parameters for the Cu atom are those included in ICONC&INPUTC.[24]
The geometry was kept fixed for all the calculations. Selected bond distances and angles for the more stable structure used for the calculations are reported in the supplementary material.
In order to simplify the MO analysis of the complex molecules the C(5), C(6), C(7), and C(8) atoms of piroxicam (Scheme 1) were removed; the valences of C(9) and C(10) (linked each other by a double bond) were saturated by H atoms. DMF molecules were replaced by formamide molecoules.

### Acknowledgments
We thank Professor Luigi G.Marzilli, Emory University, for helpful comments, and Pfizer Italia (Roma) for the generous supplying of piroxicam.

### Supplementary material
A table of experimental carbon chemical shift, and atomic charges and s population (Extended-Huckel) of the atoms of HPir and Pir$^-$. A table of selected geometrical parameters for the calculated (MM) and observed (X-Ray) structures.

### References
1. K.D.Karlin, Z.Tyeklar, in *Models in Inorganic Chemistry, Ad. Inorg. Biochem.*, G.L.Eichhorn and L.G.Marzilli, Eds., PTR Prentice Hall, Englewood Clifs, New Jersey, 1993, **9**, Ch. 4.
2. (a) A.R.Cross and O.T.G.Jones, *Biochim. Biophys. Acta*, 1991, **1057**, 281; (b) C.Muscari, M.Frascaro, C.Guarnieri, and C.M.Caldarera, *Biochim. Biophys. Acta*, 1990, **1015**, 200.
3. (a) J.A.Tainer, E.D.Getzoff, K.M.Beem, J.S. Richardson, and D.C.Richardson, *J.Biol.Med.*, 1982, **160**, 181; (b) J.A.Tainer, E.D.Getzoff, J.S.Richardson, and D.C.Richardson, *Nature*, 1983, **306**, 284.
4. M.Sette, M.Paci, A.Desideri, and G.Rotilio, *Biochemistry*, 1992, **31**, 12410.
5. J.R.J.Sorenson, *Prog.Med.Chem.*, 1989, **26**, 437.
6. (a) J.R.J.Sorenson, *J.Med.Chem.*, 1976, **19**,135; (b) A.E.Underhill, A.Bury, R.J.Odling, M.B.Fleet, A.Stevens, and P.S.Gomm, *J. Inorg. Biochem.*, 1989, **37**, 1.
7. R.Cini, G.Giorgi, A.Cinquantini, C.Rossi, and M.Sabat, *Inorg. Chem.*, 1990, **29**, 5197.
8. S.J.Beveridge, W.R.Walker, and M.W.Whitehouse, *J.Pharm. Pharmacol.*, 1980, **32**, 425.
9. (a) L.G.Sillén and A.E.Martell, *Stability Constant of Metal-Ion Complexes, Supplement No 1*, The Chemical Society, Burlington House, London, **1971**; (b) *Critical Stability Constants*, A.E.Martell and R.M.Smith, eds., Plenum Press, New York, **1974**, Vol. 1; (c) *Critical Stability Constants*, A.E.Martell and R.M.Smith, eds., Plenum Press, New York, **1977**, Vol. **3**.
10. M.Wurl, H.Fritsch, and R.Muller Peddinghaus, *Arzeneimittelforschung.*, 1987, **37**, 614.
11. (a) B.Kojić-Prodić, and Z.Ružić-Toroš, *Acta Cryst.*, 1982, **B38**, 2948; (b) I.H.Suh, K.J.Kim, T.S.Ko, and B.H.Kim, *Chungnam J. Sci.* (Eng.), 1989, **16**, 30.

12. J.Bordner, P.D.Hammen, and E.B.Whipple, *J.Am.Chem.Soc.*, 1989, **111**, 6572.
13. J.Bordner, J.A.Richards, P.Weeks and E.B.Whipple, *Acta Cryst.*, 1984, **C40**, 989.
14. J.M.Geckle, D.M.Rescek, and E.B.Whipple, *Magnetic Resonance in Chemistry*, 1989, **27**, 150.
15. J.K.Kochi, *J.Am.Chem.Soc.*, 1955, **77**, 5274.
16. (a) B.J.Hathaway and D.E.Billing, *Coord.Chem.Rev.*, 1970, **5**, 143; (b) B.J.Hathaway and A.A.G.Tomlinson, *Coord.Chem.Rev.* , 1970, **5**, 1.
17. (a) A.G.Massey in *Comprehensive Inorganic Chemistry*, Eds. J.C.Bailar, H.J.Emeléus, R.Nyholm, and A.F.Trotman-Dickenson, Pergamon, Oxford, **1973**, Ch. 27, Vol. **3**, and references therein; (b) F.A.Cotton and G.Wilkinson, *Advanced Inorganic Chemistry*, Wiley-Interscience, New York, **1988**.
18. (a) G.Moro and J.H.Freed *J. Chem. Phys.*, 1981, **74**, 3757; (b) G.Della Lunga, R.Pogni, and R.Basosi, *J.Phys. Chem.*, 1994, **98**, 3937.
19. (a) G.Rakhit, W.E.Antholine, W.Froncisz, J.S.Hyde, J.R.Pilbrow, G.R.Sinclair, and B.Sarkar *J.Inorg.Biochem.*, 1985, **25**, 217; (b) R.Pogni, G.Della Lunga, and R.Basosi *J.Am.Chem.Soc.*, 1993, **115**, 1546.
20. (a) A.Allerhand and R.A.Komoroski, *J.Am.Chem.Soc.*, 1973, **95**, 8228; (b) R.S.Norton and A.Allerhand, *J.Am.Chem.Soc.*, 1976, **98**, 1007.
21. N.L.Allinger, *J.Am.Chem.Soc.*, 1977, **99**, 8127.
22. (a) L.Hansen, R.Cini, A.Taylor, Jr., and L.G.Marzilli, *Inorg.Chem.*, 1992, **31**, 2801; (b) K.R.Adam, B.J.McCool, A.J.Leong, L.F.Lindoy, C.W.Ansel, P.J.Baillie, K.P.Dacey, L.A.Drummond, K.Hedrick, and M.McPartlin, *J.Chem.Soc., Dalton Trans.*, **1990**, 3435; (c) D.A.Buckingham, P.J.Creswell, R.J.Dellaca, M.Dwyer, G.L.Gainsford, L.G.Marzilli, I.E.Maxwell, W.T.Robinson, A.M.Sargeson, and K.R.Turnbull, *J.Am.Chem.Soc.*, 1974, **96**, 1713.
23. W.C.Still, F.Mohamadi, N.G.J.Richards, W.C.Guida, M.Lipton, G.Chang, T.Hendrickson, F.DeGunst, and W.Hasel, *MacroModel V3.0*. Department of Chemistry, Columbia University, New York, **1990**.
24. G.Calzaferri and M.Brändle, *ICONC&INPUTC*, University of Berne, **1992**, QCPE program No QCMP 116.
25. (a) G.Calzaferri, L.Forss, and I.Kamber, *J.Phys.Chem.*, 1989, **93**, 5366; (b) G.Calzaferri and R.Hoffmann, *J.Chem.Soc., Dalton Trans.*, **1991**, 917; (c) ICONC&INPUTC documentation (see Ref. 24).
26. C.K.Johnson, *ORTEPII, Rep. ORNL-3794*, Oak Ridge National Laboratory, TN, USA, **1971**.

**Received: July 27, 1994 - Accepted: August 30, 1994 - Received in camera-ready format: September 20, 1994**

